# Family Dynamics and Grandparents' Anxiety and Depression in Intergenerational Rearing Families: A Correlational Study

**DOI:** 10.3389/fpsyt.2021.633773

**Published:** 2021-05-20

**Authors:** Chun-Yan Yang, Hong-Jiao Xu, Shan-Shan Liu, Yue-Jing Wu, Yun Long, Hong-Sheng Liu, Yao-Pian Chen, Xia Li

**Affiliations:** ^1^Geriatric Psychiatry Department, Shanghai Mental Health Center, Shanghai, China; ^2^Dalian Seventh People's Hospital, Dalian Mental Health Center, Dalian, China; ^3^Qingdao Mental Health Center, Qingdao University, Qingdao, China

**Keywords:** intergenerational rearing, anxiety, depression, correlation, family dynamics

## Abstract

**Background:** In China, intergenerational rearing is a ubiquitous phenomenon based on unique national conditions. This study aimed to explore family dynamics in intergenerational rearing families as well as their correlation with older household members' anxiety and depression.

**Methods:** The elderly from intergenerational (*n* = 141) and non-intergenerational rearing families (*n* = 266) were investigated using the following scales: the general information questionnaire, Self-Rating Scale of Systemic Family Dynamics, Geriatric Depression Scale, and Self-Rating Anxiety Scale.

**Results:** Scores from the four dimensions (family atmosphere, system logic, individuation, and the concept of disease) of the structure of family dynamics were computed. The comparison of these dimensions scores and the total scores of grandparents' anxiety and depression for the two groups were not statistically significant (*p* > 0.05). In Pearson's correlation analysis, no significant correlation between the family atmosphere dimension and the total score of the grandparents' depression and anxiety scales was observed. The system logic aspect was negatively correlated with depression and anxiety scale scores. The individual dimension was positively correlated with the anxiety scale scores. The disease concept dimension was positively correlated with depression and anxiety scale scores. Hence, the results were statistically significant.

**Conclusion:** There were no significant differences in terms of family dynamics and risk of anxiety and depression among grandparents between the two family types. The system logic, individuation, and disease concept dimensions were correlated with their anxiety and depression.

## Introduction

In China, intergenerational rearing is a ubiquitous phenomenon based on unique national conditions. The elderly participate in their grandchildren's non-age period. Meanwhile, a large number of new parenting concepts have emerged because of rapid economic development over the last 40 years, since the implementation of China's reform and opening up.

Family dynamics, a model of family categories developed by the Heidelberg Group, describe the domestic relationships and their interactive features completely and systematically. By accepting the opinions of the Milan Systemic Family Therapy Group and performing treatment on mentally ill patients based on systemic family theory, they discovered the family dynamic structural characteristics of schizophrenic, bipolar affective disorder, and psychosomatic patients. These features are related to the recovery of individuals' psychological symptoms (1). In China, Zhao et al. maintain the same outlook that family dynamics are a subject focusing on the interaction between the family's internal (psychology, behavior, and communication) and external environments, in which its members' relations are reflected. Therefore, we speculated that the family dynamic structure of intergenerational rearing families may differ from others, and these variations would have numerous noticeable effects on grandparents' mental health.

In recent years, studies on intergenerational rearing problems have received increasing attention globally, and two contrary views regarding the influence of grandchildren rearing on grandparents exist. Some research has shown that it has a positive effect on the physical and mental health of the elderly (2, 3). However, other studies have demonstrated its negative impact on grandparents; this is because the investment of time, physical strength, and emotions lead to early retirement and psychological pressures such as loneliness and trepidation (4–6).

This study investigated whether intergenerational rearing could produce different family dynamic structures, as well as the relationship between them and the elderly's anxiety and depression.

## Methodology

### Participants and Procedure

Screening and evaluation of 957 older people living in Yinhang Street Community, Yangpu District, Shanghai was performed from November 1 to December 4, 2019. In the current research, we adopted random sampling methods to obtain the sample. The survey was conducted during a routine check-up, which is a well-fare provided by the local government. Among all the participants, there are 407 of them completed the questionnaires (141 from intergenerational family and 266 from non-intergenerational family). The participants were required to be over 55 years old, have normal vision (naked eye or redness), and have the ability to communicate. Informed consent was obtained from them prior to their participation in the survey. The exclusion criteria included having a history of major physical illness, particularly those that may be associated with alterations in brain tissues, as well as abnormalities in the nervous system, including major head traumas (loss of consciousness lasting over 5 min), epilepsy, cerebrovascular diseases, brain tumors, and neurodegenerative diseases. The study protocol was approved by the Ethics Committee of the Shanghai Mental Health Center subsequent to the initiation of the research.

### Instruments

We used the Self-Rating Scale of Systemic Family Dynamics (SSFD) to explore family dynamics, the Geriatric Depression Scale (GDS) to determine the participants' depressive states, and the Self-Rating Anxiety Scale (SAS) to assess their anxiety states.

#### The Self-Rating Scale of Systemic Family Dynamics (SSFD)

The SSFD comprises 29 items categorized into four dimensions. First, family atmosphere refers to the emotional aspect of communication within the family system (7). A higher score indicates hostility and oppression, while a lower score reflects pleasantness and comfort. Second, system logic entails the logical characteristics of household members' value judgments. A high score indicates resolution, while a lower one reflects indecisiveness. Third, personalization is the differentiation of their emotions and behaviors. Lower scores indicate that a family member is more independent. Last, disease concept evaluates the amount of responsibility the members consider they ought to shoulder when having a disease. A high score indicates that they believe themselves to be absolute victims, whereas a low score means that they perceive themselves as complete actors. Each item is marked from 1 (completely suitable) to 5 (completely wrong) the Cronbach coefficients of the SSFD are 0.74 for “family atmosphere,” 0.79 for “system logic,” and 0.81 for “disease cognition,” respectively.

#### The Geriatric Depression Scale (GDS)

The GDS (8), which is aims at depression assessment of elderly people aged over 56 years, was developed in 1982 by Brank et al. It evaluates their most relevant feelings over a 1-week period, and contains 15 columns. The scores reflect the state of depression, with 0–4 points referred to as normal, and 5–8, 9–11, and 12–15 points as mild, moderate, and major depression, respectively. The Cronbach's coefficient is 0.64 for GDS in the current research.

#### The Self-Rating Anxiety Scale (SAS)

The 20-item SAS is a self-rating measure including 20 items (9). It evaluates the user's subjective feelings during the last week, and the scores are divided into 4 degrees. The first, second, third, and fourth levels refer to no, sometimes yes, frequently yes, and almost yes or yes, respectively. The cut-off score of 41 may indicate an anxiety condition. The Cronbach's coefficient is 0.77 for SAS in the current research.

### Survey Method

The survey comprised an on-site questionnaire conducted by several professional evaluators who underwent a standardized training. They briefly explained the questionnaire through standard instructions prior to the survey; it was later completed by the participants independently. The evaluators provided appropriate explanations to those individuals who had any related queries. All the survey forms were collected immediately; the data were checked, and entered using Epidata version 3.1; a logical test was performed in order to exclude invalid questionnaires.

### Statistical Analyses

The Statistical Package for the Social Sciences version 17.0 software was used for data analysis, and the measured data were expressed as mean ± standard deviation (*x* ± s). Comparisons between groups were assessed using *t*-tests. The association of anxiety and depression with the dimensions of family dynamic structure was investigated using Pearson correlation analysis. Statistical significant was set at *p* < 0.05.

## Results

The mean ages of the intergenerational and the non-intergenerational rearing group were 65.35 ± 5.00 and 65.21 ± 8.36, respectively. The former comprised 53 males and 88 females, while the latter included 102 males and 164. Neither group showed any significant differences in gender, as shown in Table 1.

**Table 1:** Socio-demographic data from the participant grandmothers. *n* (%).

**Variable**	**Intergenerational rearing families (*n* = 141)**	**Non-intergenerational rearing families (*n* = 266)**	**t/χ^2^**	***p-value***
Age	65.35 ± 5.00	65.21 ± 8.36	0.18	0.86
Gender			0.022	0.881
Males	53 (37.59)	102 (38.35)		
Females	88 (62.41)	164 (61.65)		

*P*-value > 0.05 (see Table 2). Scores of family dynamics (family atmosphere, system logic, individuation, and the concept of disease), anxiety and depression were computed. The results from *T*-tests showed that the inter-generational rearing group did not differ significantly from the non-inter-generational group on the aforementioned variables (*p* > 0).

**Table 2:** The comparison of dimensions scores of family dynamics, total scores of grandparents' anxiety-depression in such two kinds of families. (x̄ ± s).

**Scales**	**Intergenerational rearing families (*n* = 141)**	**Non-intergenerational rearing families (*n* = 266)**	***t***	***p-value***
**Family dynamic structure**				
Family atmosphere	26.79 ± 6.15	27.41 ± 5.20	−1.08	0.282
The system logic	15.82 ± 4.84	15.85 ± 5.01	−0.05	0.959
Individuation	18.01 ± 4.71	18.02 ± 3.96	−0.02	0.985
The concept of disease	8.41 ± 3.44	8.59 ± 3.29	−0.53	0.600
**Depression scale and the anxiety scale**				
GDS	3.13 ± 3.00	3.01 ± 2.89	0.38	0.703
SAS	33.30 ± 6.58	33.01 ± 6.86	0.42	0.673

Pearson's correlation analysis revealed no significant correlation between the family atmosphere dimension and the total score of the grandparents' depression and anxiety scales; however, the system logic aspect was negatively correlated with it. The individual dimension had a positive correlation with the anxiety scale score. Furthermore, the disease concept element was positively correlated with the scores on the depression and anxiety measures. Results were listed in Table 3.

**Table 3:** Pearson's correlation between the dimensions scores of family dynamics and total scores of grandparents' anxiety-depression. (*r*).

	**GDS**	**SAS**
Family atmosphere	0.012	0.079
The system logic	−0.162^**^	−0.155^**^
Individuation	0.039	0.103^*^
The concept of disease	0.127^*^	0.145^**^

* *P < 0.01*.

** *P < 0.05*.

## Discussion

Family is a basic environment that shapes personality, the constituent factors of which influence individual personality performance and psychological health (10). Family intimacy is an index that evaluates the emotional connection between household members, and describes various family categories. Theoretically, a balanced family intimacy is ideal. Overly intimate or distant relationships are harmful to an individual's mental health in several ways. The former entangle family members' lives excessively, while the latter estrange them (11).

Family dynamics provide an effective observational perspective for clinical practice by focusing on emotions, behaviors, negotiations, and their processes within the family, as well as by abstracting the psychological processes and interpersonal interaction patterns of the family (12, 13). Moreover, it is an indicator that can comprehensively assess the family system (14).

This study investigated the family dynamic structural features of intergenerational and non-intergenerational rearing families and their association with the anxiety and depression emotional characteristics of grandparents. The results revealed no significant differences in the family dynamic features and anxiety and depression between the two groups. The systemic logical dimension was inversely related to the total score of the depression and anxiety scales. The disease concept dimension was positively correlated with the total score of the depression and anxiety scales. The lower the score on this dimension, the lower the scores on the two scales. If elderly family members link disease and rehabilitation to self-response and subjective effort instead of regarding themselves as victims of diseases, depression and anxiety are unlikely to occur.

A study conducted in China reported that older people rearing their grandchildren were limited by factors such as age, physical health, education level, economic conditions, urban, and rural environments. The previous findings regarding how inter-genenerational rearing may influence the mental health conditions are mixed (15, 16). Research on the impact of intergenerational rearing on aging individuals found that raising grandchildren can benefit their psychological health, reinforce the intergenerational relationships of the rural elderly, and protect their cognitive functions (17–19). Similarly, a Taiwanese study indicated that intergenerational rearing can decrease the risk of depression and anxiety in aging individuals (20). On the other hand, research holding the opinion that it has a negative impact on older people found that it can cause them various psychological problems. Reliance on grandchildren can influence the grandparents' mental health and result in early retirement (21–23). Neither of the two perspectives has been proven in this research and thus further research efforts are needed.

This research was unable to clarify the differences in the family dynamic structure between intergenerational and non-intergenerational rearing and the impact of the former on the elderly, the current study provided evidence for an association between the family dynamic structure and the elderly's depression and anxiety. The influence of family on the psychological health of the elderly cannot be disregarded because it is the most important living environment for aging individuals. Intergenerational rearing problems need to be considered in different respects In the future, more research effort should focus on the relations between family dynamics and elderly people's mental health conditions. Limitations As the research focused on the elderly, for whom the concepts of family dynamics were relatively abstract, it was challenging to avoid the occurrence of bias in their responses. This resulted in some interference with the findings. The representativeness of this research is limited because the sample comprised elderly individuals belonging to the same community. Therefore, to improve the investigation, the sample needs to be extended to different areas, and the quality of the survey questionnaires should be strictly controlled. Additional targeted research conclusions will be explored after an in-depth analysis of the results.

## Data Availability Statement

The raw data supporting the conclusions of this article will be made available by the authors, without undue reservation.

## Ethics Statement

The studies involving human participants were reviewed and approved by Ethics committee of Dalian No. 7 People's Hospital. The patients/participants provided their written informed consent to participate in this study.

## Author Contributions

XL proposed the research idea. CY-Y collected the data and wrote the paper. H-JX, S-SL, Y-JW, YL, H-SL, and Y-PC helped in data collection, revised the manuscript, and provided many constructive suggestions. All authors contributed to the article and approved the submitted version.

## Conflict of Interest

The authors declare that the research was conducted in the absence of any commercial or financial relationships that could be construed as a potential conflict of interest.
